# Number of human protein interactions correlates with structural, but not regulatory conservation of the respective genes

**DOI:** 10.3389/fgene.2024.1472638

**Published:** 2024-10-29

**Authors:** Rijalda Mekic, Marianna A. Zolotovskaia, Maksim Sorokin, Tharaa Mohammad, Nina Shaban, Ivan Musatov, Victor Tkachev, Alexander Modestov, Alexander Simonov, Denis Kuzmin, Anton Buzdin

**Affiliations:** ^1^ Laboratory for Translational Genomic Bioinformatics, Moscow Institute of Physics and Technology, Dolgoprudny, Russia; ^2^ Moscow Center for Advanced Studies, Moscow, Russia; ^3^ Laboratory of Bioinformatics, Endocrinology Research Center, Moscow, Russia; ^4^ Laboratory of Clinical and Genomic Bioinformatics, I. M. Sechenov First Moscow State Medical University, Moscow, Russia; ^5^ Laboratory of Systems Biology, Shemyakin-Ovchinnikov Institute of Bioorganic Chemistry, Moscow, Russia; ^6^ Oncobox Ltd., Moscow, Russia; ^7^ PathoBiology Group, European Organization for Research and Treatment of Cancer (EORTC), Brussels, Belgium

**Keywords:** human gene regulation, molecular evolution, retrospect, regulatory evolution rate, structural evolution rate, human interactome model, molecular pathways, oncoboxpd database

## Abstract

**Introduction:**

The differential ratio of nonsynonymous to synonymous nucleotide substitutions (dN/dS) is a common measure of the rate of structural evolution in proteincoding genes. In addition, we recently suggested that the proportion of transposable elements in gene promoters that host functional genomic sites serves as a marker of the rate of regulatory evolution of genes. Such functional genomic regions may include transcription factor binding sites and modified histone binding loci.

**Methods:**

Here, we constructed a model of the human interactome based on 600,136 documented molecular interactions and investigated the overall relationship between the number of interactions of each protein and the rate of structural and regulatory evolution of the corresponding genes.

**Results:**

By evaluating a total of 4,505 human genes and 1,936 molecular pathways we found a general correlation between structural and regulatory evolution rate metrics (Spearman 0.08–0.16 and 0.25–0.37 for gene and pathway levels, respectively, *p* < 0.01). Further exploration revealed in the established human interactome model lack of correlation between the rate of gene regulatory evolution and the number of protein interactions on gene level, and weak negative correlation (∼0.15) on pathway level. We also found a statistically significant negative correlation between the rate of gene structural evolution and the number of protein interactions (Spearman −0.11 and −0.3 for gene and pathway levels, respectively, *p* < 0.01).

**Discussion:**

Our result suggests stronger structural rather than regulatory conservation of genes whose protein products have multiple interaction partners.

## 1 Introduction

The structural evolution of protein-coding genes is currently central to the field of evolutionary genomics. An established method for quantifying the magnitude of evolutionary pressure on protein-coding genes includes estimation of the ratio of nonsynonymous (changing amino acids) to synonymous (not changing) base substitutions in coding codons, known as dN/dS (Jeffares et al., 2015). Specifically, a dN/dS ratio significantly greater than one serves as an indicator of positive Darwinian selection, while a ratio below one indicates purifying selection. In turn, a dN/dS ratio equal or close to one indicates neutral selection. Thus, a higher dN/dS value corresponds to an accelerated rate of gene structural evolution, while a lower value reflects structural conservation of a gene product (Kryazhimskiy and Plotkin, 2008).

Another important aspect of gene evolution is related to functional changes in transcriptional activity. Recently, we have proposed for the first time a *Retrospect* method that measures the rate of regulatory evolution of genes through relative quantification of the enrichment of gene promoters with functional motifs that map to transposable elements. Such functional motifs can be transcription factor binding sites or modified histone binding loci. Greater association of regulatory modules with transposable elements means faster evolution of gene regulation. In the case of the human genome, Retrospect considers a class of transposable elements called retroelements (REs), which are selfish elements capable of replicating in the genome through reverse transcription (Nikitin et al., 2019C).

REs constitute the vast majority of human transposable elements and occupy about 40% of total human DNA. They can participate in the control of gene expression by providing functional regulatory elements such as alternative promoters, enhancers, silencers, polyadenylation signals and others (Gogvadze and Buzdin, 2009). In particular, about half of all transcription factor binding sites (TFBS) in the human genome are estimated to be associated with REs (Nikitin et al., 2019a). In addition, REs may be involved in chromatin tag rearrangement by converting euchromatic (active) regions to heterochromatic (inactive) regions and *vice versa*. In general, RE insertions tend to be much less conserved than surrounding genomic regions (Lander et al., 2001) and thus may indicate rapidly evolving regulatory features if they are enriched with functional motifs.

The regulatory influence of REs on individual genes can be measured using a metric called *Gene RE-linked Enrichment score (GRE)* (Nikitin et al., 2018). This metric can be applied to different types of regulatory elements such as TFBS or chromatin tags. For example, in the case of TFBS analysis, a gene’s GRE score is calculated as the sum of RE-linked TFBS hits mapped in the 10-kb neighborhood of its transcription start site divided by the average number of RE-linked TFBS hits across all genes analyzed. A further modification of this metric, called Normalized Gene RE-linked Enrichment score (NGRE), takes into account the fact that the number of TFBS hits, whether RE-linked or not, can vary greatly between different genes with different regulatory mechanisms. Thus, an NGRE estimate for a gene can be obtained by further normalizing the GRE value by the balanced number of all (not just RE-linked) TFBS hits for that gene. Similarly, GRE and NGRE scores can also be calculated for chromatin tags such as modified histone binding sites (Igolkina et al., 2019).

Another approach is to look at the bigger picture by combining genes at the molecular pathway level. The quantitative measure here is the pathway involvement index (PII), calculated as the average GRE value for all genes involved in the pathway of interest. Similarly, PII can be normalized by the average effect of TFBS or chromatin tags on all genes in the pathway analyzed, yielding a normalized pathway involvement index (NPII). In general, aggregating data at the molecular pathway level improves the overall stability of the data by reducing the bias that can be caused by variations in metrics for individual genes (Borisov et al., 2017).

Previously, for the first time we reported a consistent positive correlation between the rate of structural and regulatory evolution of human protein coding genes and molecular pathways (Zakharova et al., 2023).

In addition, the pathway-level approach can be transferred to the interactome level. In particular, many attempts have been made to investigate the relationship between the evolution of proteins and their properties in the interaction network, such as connectivity. Indeed, it has been shown that the rate of structural evolution of individual proteins is negatively correlated with the number of their interactions in yeast (Fraser et al., 2002). Indeed, intuitively one can expect that genes encoding proteins involved in a large number of molecular interactions should be more evolutionarily conservative, since excessive structural variation in them is more likely to disrupt some of their functional sites that support their downstream interactions. However, this conclusion has been questioned (Bloom and Adami, 2003; Jordan et al., 2003; Batada et al., 2006), and a number of studies (Hahn et al., 2004; Drummond et al., 2006) have reported that the relationship between knot connectivity and evolutionary rate is weak, although significant.

However, to the best of our knowledge, there have been no studies to date examining the regulatory evolution of protein-coding genes in the context of interactome connectivity.

In this paper, we combined the quantitative measures of structural and regulatory evolution described above to characterize the human interactome. We constructed a model of the human interactome based on 600,136 documented molecular interactions and investigated the overall correlation between the number of interactions of each protein and the rate of structural and regulatory evolution of the corresponding genes. We found a general correlation between metrics for the rate of structural and regulatory evolution of genes encoding human proteins for both transcription factor binding sites and histone modification mapping data.

However, no correlation was observed between the rate of regulatory evolution and the number of gene interactions in the human interactome model. In contrast, we found a negative correlation between the rate of structural evolution and the number of gene interactions in the human interactome model, which was more pronounced for the pathway level of data analysis. Taken together, these results suggest structural rather than regulatory conservation of genes whose protein products have multiple interaction partners.

## 2 Methods

### 2.1 Genomic retroelement enrichment data

To quantitatively characterize the rates of regulatory evolution of genes, we have previously introduced analytic metrics termed *Gene RE-linked Enrichment* (*GRE*) and *Normalized RE-linked Enrichment* (NGRE) (Nikitin et al., 2018). The GRE score of a gene *x* characterizes the total number of RE-linked regulatory elements in that gene and is calculated as follows:

$${GRE}_{x}=\frac{{FES}_{x}}{\frac{1}{n}\sum_{i=1}^{n}{FES}_{i}},$$

where *FESx* (Feature Enrichment Score) is the number of RE-linked regulatory element alignment hits that were mapped within 10 kb-frame centered at the canonical transcription start site of gene *x; n* is the total number of genes under analysis, and the denominator is the average FES of all genes under analysis. GRE score is calculated for a single type of a regulatory feature at a time, e.g., for mapped hits of TFBS or of a specific histone tag.

**FIGURE 1:**
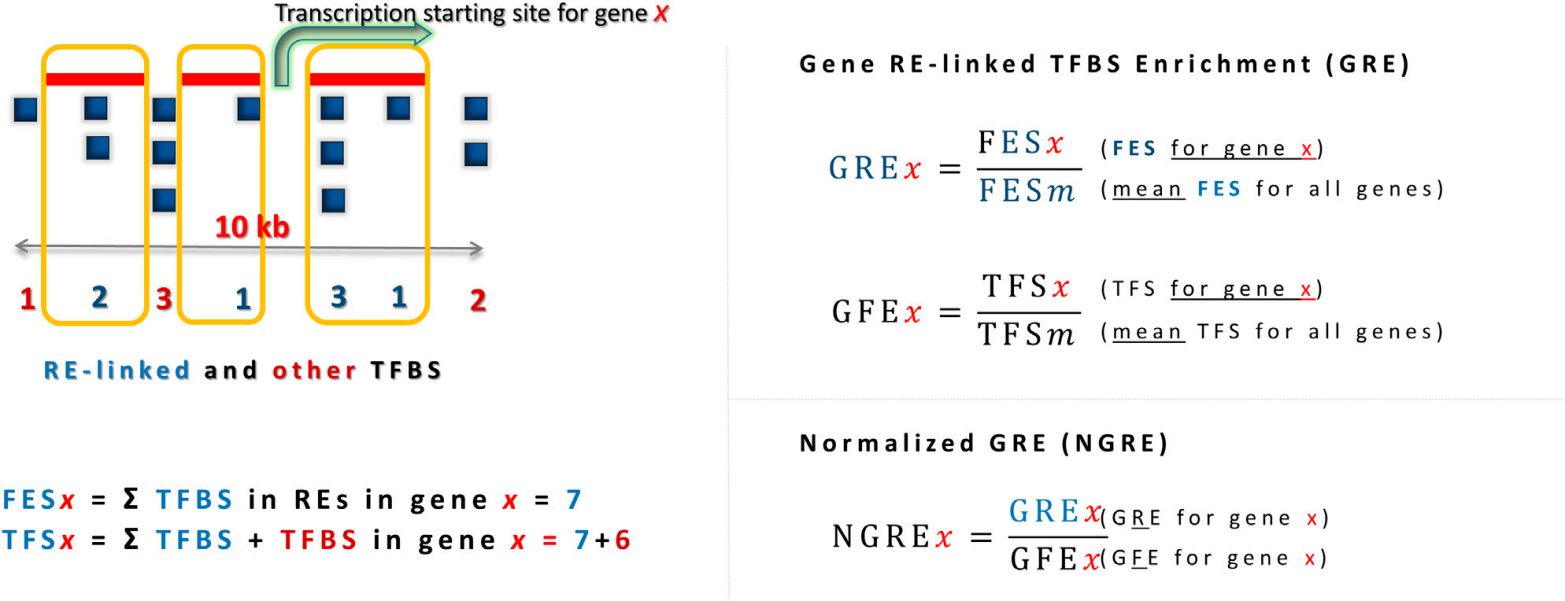
Characterization of regulatory evolution rate at gene-wise level, exemplified for the analysis of TFBS data. Higher GRE reflects greater *number* of RE-linked TFBS in a gene, higher NGRE - greater *proportion* of RE-linked TFBS in a gene. RE-linked TFBS are boxed. *TFS*_*x*_ (Total Feature Score) is the total number of hits associated with the regulatory feature of interest (both RE-linked and not) mapped within a 10-kb vicinity of the transcription start site of a gene *x*.

Additional variable GFE (gene feature enrichment) score characterizes gene-specific hits distribution trends, expressed by the formula:

$${GFE}_{x}=\frac{{TFS}_{x}}{\frac{1}{n}\sum_{i=1}^{n}{TFS}_{i}},$$

where TFSx is the total number of feature hits mapped in the 10-kb neighborhood of gene x and TFSm is the mean TFS for all genes under investigation (Figure 1).

A normalized RE-specific enrichment measure for an individual gene termed NGRE (normalized gene RE-linked enrichment score) was calculated for a gene *x* as follows:

$${NGRE}_{x}=\frac{{GRE}_{x}}{{GFE}_{x}},$$

Relative retroelement enrichment scores for genes (NGRE), which were previously calculated using ChIP-seq profiles of human cell lines, were extracted from our previous published datasets (Igolkina et al., 2019; Nikitin et al., 2019B) for six histone modifications (H3K4me1, H3K4me3, H3K9ac, H3K27ac, H3K27me3, and H3K9me3), and for a total of 563 transcription factor binding sites (TFBSs) in 13 human cell lines (Nikitin et al., 2019a). NGRE reflects the ratio of RE-linked regulatory elements to all regulatory elements in the 10-kb frame centered around the gene canonical transcriptional start site (Nikitin et al., 2018; 2019c; 2019a; Igolkina et al., 2019).

For the analysis at the level of molecular pathways, the *Normalized Pathway Involvement Index (NPII)* that describes the *normalized proportion* of RE-associated regulatory features of a given type in pathway member genes, was calculated as follows:

$${NPII}_{p}=\frac{1}{k}\sum_{j=1}^{k}N{GRE}_{j},$$

where *NGREj* is the NGRE of a gene *j* involved in a pathway *p*, and *k* is the total number of genes in this pathway.

### 2.2 Assessment of structural and functional evolution rates

For the analysis of *structural evolution*, at the gene-wise level we used dN/dS values for 10,890 common hominid genes extracted from (Scally et al., 2012). For the pathway level of data analysis, we used averaged dN/dS_pw values calculated across all genes participating in the respective pathway, according to our previously published research (Zakharova et al., 2023).

For the analysis of *regulatory evolution*, we used aggregated NGRE (gene-wise level) and NPII (for pathways) scores. NGRE and NPII were aggregated separately for the different types of biomarkers used: (*i*) TFBS, (*ii*) active, and (*iii*) condensed chromatin marks. Pre-calculated GRE and NGRE values for 10,891 genes in five human cell lines (K562, HepG2, GM12878, MCF-7, HeLa-s3) were taken from our previous studies: for TFBS data (Nikitin et al., 2019b; Zakharova et al., 2023), for H3K4me1 chromatin mark (Nikitin et al., 2019c), and for chromatin marks H3K4me3, H3K9ac, H3K27ac, H3K27me3, and H3K9me3 (Igolkina et al., 2019). For each type of biomarkers, weighted average NGRE profiles (NGRE_AGG_) were calculated among all 5 cell lines under analysis. The weight of a profile *i* was expressed by the formula

$$w_{i}={\left(1-z_{i}\right)}^{3}$$

where *z*_*i*_ is the proportion of zero/no data values in the profile *i*. This factor increases importance of more informative profiles in the calculation of an overall evolutionary metric.

### 2.3 Link between connectivity and evolution

We used our previously published molecular interactions database comprising known protein-protein interactions and metabolic reactions including 293,187 protein-protein and 600,136 total interactions (Zolotovskaia et al., 2022a; 2022b; 2023) to create human interactome model and quantify interactions for 7,483 protein-coding genes.

To create this model, we used molecular architectures of 50,178 different pathways from public databases, uniformly processed (Zolotovskaia et al., 2022b). Complex pathway nodes containing n molecular participants were divided into n nodes with only one participant. Thus, each vertex represents one pathway participant on the graph. We then combined all pathway graphs together based on the coinciding gene products and metabolites.

From all these pathways, we excluded molecular participants which were not connected within the overall network (less than 1% of the initial pathway members). The remaining molecular interactors formed a connected graph.

The model exists as a directed graph where the nodes correspond to gene products and metabolites, and edges represent known pairwise molecular interactions between the nodes (Supplementary Table S1). To assess connectivity on both gene and pathway levels, we utilized data from interactome and OncoboxPD pathway database (Zolotovskaia et al., 2022b; Zakharova et al., 2023). The connectivity of a protein-coding gene was defined as the number of incoming and outcoming edges for the corresponding node. For the comparison of gene connectivity with the NGRE scores and dN/dS, we used intersected gene set where both types of data (connectivity and evolution metrics), were available (Supplementary Figure S1). 12 genes were excluded as outliers by connectivity (Supplementary Figure S2). We obtained 4,505 genes (Supplementary Table S2). The same gene sets were used also for the analysis at the level of molecular pathways (Supplementary Figure S1).

The metric for assessing the connectivity of molecular pathways is the averaged number of interactions per pathway. However, the interactions were taken not from the individual pathway graph, but they were obtained from the reconstructed human whole-interactome model. The number of interactions was then divided by the number of respective genes present in the corresponding pathway. Thus, a normalized measure of interactions per pathway was obtained, which provided an estimate of the interconnectivity and interactions of genes in molecular pathways.

We evaluated connectivity in two ways: considering direct protein-protein interactions and considering all interactions including direct and indirect interactions with proteins, metabolites. Indirect interactions were represented by interactions through auxiliary nodes of biochemical reactions and transport processes.

### 2.4 Estimating evolutionary rate of molecular pathways

To analyze pathways and estimate their evolutionary rate, we algorithmically constructed molecular pathways based on a model of the human interactome that integrates protein-protein interactions and metabolic reactions (Zolotovskaia et al., 2022b; 2023).

Each molecular pathway was defined by a central gene and its immediate neighbors directly connected in the graph of the interactome. If a neighbor represented a node involved in a known biochemical reaction or transport process, all members of that process were included to maintain process integrity. This resulted in 7,483 molecular pathways. In addition, we took 3,025 classic molecular pathways from OncoboxPD database (Zolotovskaia et al., 2022b).

10,244 of 10,508 pathways contain genes with evolution and interactome data (from the set of 4,505 genes). Then we selected pathways with 10 and more genes and with more than 60% of genes with data available to increase statistic robustness and assess objectively whole molecular pathways. Also, we excluded pathways, which were full duplicates accordingly to their gene composition and pathways with high similarity (Jaccard coefficient >0.7). This resulted in 1936 molecular pathways. All filtration steps are available in Supplementary Figure S1. Duplicated and similar pathways were excluded to avoid false positive results in further correlation analysis due to duplicated values caused by the same or similar gene composition.

For each pathway, structural evolutionary rates were determined by calculating the dN/dS_pw, that is an aggregated dN/dS value for all genes involved in a pathway, averaged to the number of gene products in a pathway, for which dN/dS data were available.

For regulatory evolution, *normalized pathway involvement indexes* (NPII) that characterize the regulatory evolution were calculated based on NGRE values. NPII values were then calculated for each type of data, and weighted average NPII values were obtained for aggregated TFBS, active and inactive chromatin tags according to (Zakharova et al., 2023).

## 3 Results

### 3.1 Design of the study

Using the Retrospect method (Nikitin et al., 2019c), we separately calculated NGRE and NPII scores for sets of (*i*) TFBS data, (*ii*) active chromatin tags, and (*iii*) inactive chromatin tags (Zakharova et al., 2023). Here, *H3K4me3*, *H3K4me1*, *H3K9ac* and *H3K27ac* histone modifications were recognized as signatures of promoter/enhancer regions and, consequently, marks of the active chromatin. Conversely, *H3K27me3* and *H3K9me3* were considered as markers of heterochromatin representing transcriptionally silent domains of DNA (Zakharova et al., 2023). In addition, *TFBS* patterns were indicative of the transcriptional factor binding regulation of genes. We then aggregated the results for each of these groups of raw data and compared the resulting aggregated NGRE_agg_ and NPII_agg_ scores with dN/dS data (gene-wise as well as aggregated at the molecular pathway level).

Using molecular interaction databases, we reconstructed the human interactome, which includes 600,136 total and 293,187 direct protein-protein interactions (Zolotovskaia et al., 2022b) and determined the number of incoming and outgoing interactions for each involved protein. Finally, we compared the obtained estimates of NGRE (gene-level *regulatory evolution* metric), NPII (pathway-level *regulatory evolution* metric), and dN/dS (gene- and pathway-level *structural evolution* metric) with the number of interactions for individual genes or molecular pathways.

### 3.2 Human oncointeractome model

Presenting overall interactome as a network/graph in addition to providing a useful option for visualization also enables applying mathematical apparatus for graph analysis, such as vertex/node degree, degree distribution, degree sequence, and Brook’s and Vizing’s theorems (Frieze et al., 1988; Karloff, 1989; Misra and Gries, 1992) Here, a vertex/node is the fundamental unit of which graphs are formed (Perfect, 1977).

**FIGURE 2:**
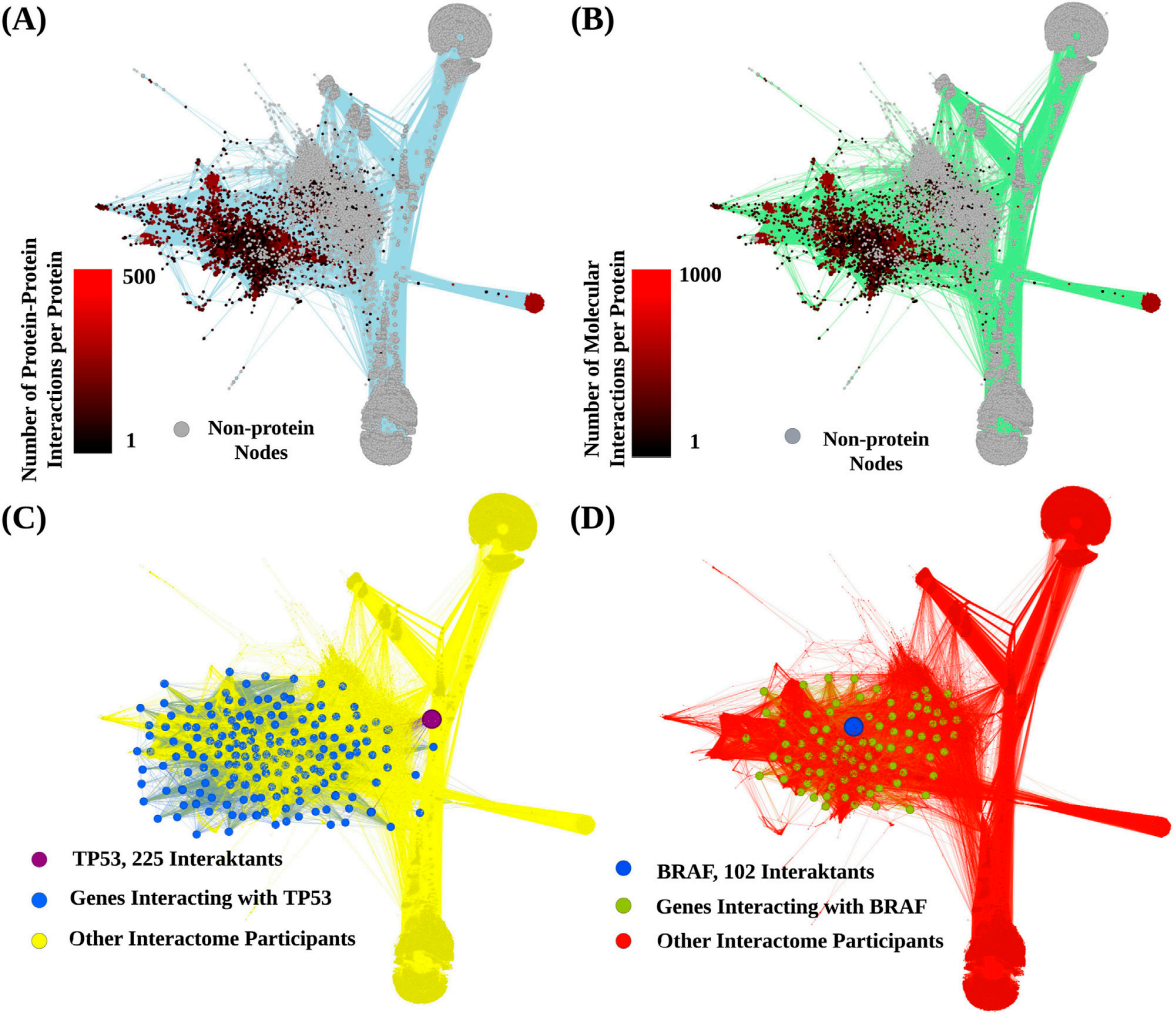
Schematic representation of the human interactome model. Graph vertices correspond to gene products, metabolites and auxiliary nodes denoted biochemical reactions and transport processes. Edges present interactions between nodes. **(A)** Color indicates number of *protein-protein interactions* per each protein node. **(B)**, Color indicates number of *total molecular interactions* per each protein node. **(C)**, representative interaction network shown for protein P53. **(D)**, representative interaction network shown for protein BRAF. Edges inherit color from the corresponding outcoming donor nodes.

In our study, we constructed a model of the human interactome (Figure 2) built with 7,483 genes accordingly to (Zolotovskaia et al., 2022b). The total number of interactions was 600,136, of which 293,187 were direct protein-protein interactions. The number of interactions for each gene product is available in Supplementary Table S2.

Some gene products showed an outstanding connectivity. For example, gene *PIK3CA* that regulates key cellular processes including growth and survival had 593 interactions. Gene *PRKACA*, known for its multifaceted role in mediating cAMP signaling and thereby influencing multiple cellular functions, had 588 mapped interactions. Similarly, gene *GNG12* that encodes an integral component of heterotrimeric G-proteins critical for signal transduction, showed 574 interactions.

In addition, some gene products had numerous connections with both proteins and metabolites, such as Lipin 1 (LPIN1), 1-Acylglycerol-3-Phosphate O-Acyltransferase 1 (AGPAT) and Phosphatidylserine Synthase 1 (PTDSS1), had 1,234, 1,227, and 960 interactions, respectively.

### 3.3 Comparison of structural and regulatory evolution rates

**FIGURE 3:**
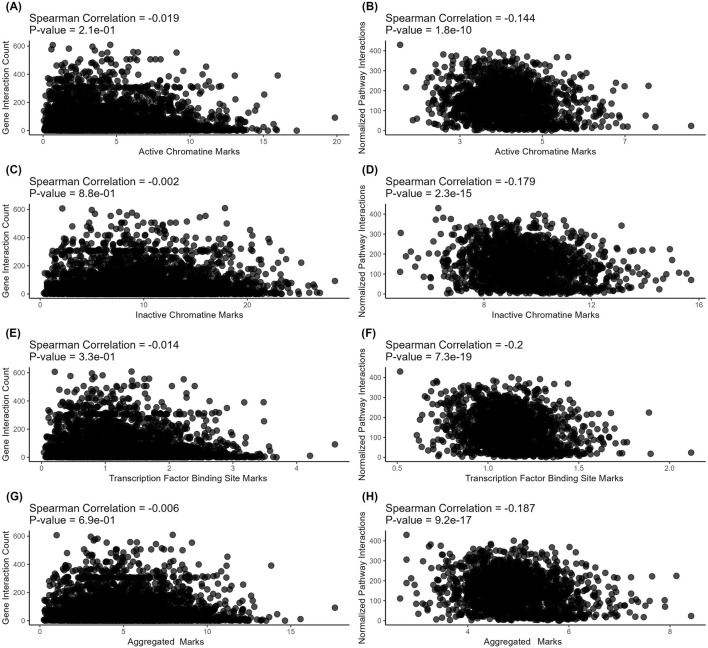
Correlation analysis of gene-level (left) and pathway-level (right) connectivity metrics deduced for *protein-protein* interactions with the respective *regulatory* evolution rates measured for the active chromatin, inactive chromatin, and TFBS marks. **(A)** Number of direct protein-protein interactions versus active chromatin marks-based retroelement enrichment metrics (NGRE_ac_). Each point corresponds to a gene. **(B)** Averaged number of direct protein-protein interactions per pathway versus active chromatin marks-based retroelement enrichment metrics (NPII_ac_). Each point corresponds to a pathway. **(C)** Number of direct protein-protein interactions versus heterochromatin marks-based retroelement enrichment metrics NGRE_hc_. Each point corresponds to a gene. **(D)** Averaged number of direct protein-protein interactions per pathway versus heterochromatin marks-based retroelement enrichment metrics (NPII_hc_). Each point corresponds to a pathway. **(E)** Number of direct protein-protein interactions versus TFBS-based retroelement enrichment metrics (NGRE_TFBS_). Each point corresponds to a gene. **(F)** Averaged number of direct protein-protein interactions per pathway versus TFBS-based retroelement enrichment metrics (NPII_TFBS_). Each point corresponds to a pathway. **(G)** Number of direct protein-protein interactions versus aggregated retroelement enrichment metrics NGRE_AGG_. Each point corresponds to a gene. **(H)** Averaged number of direct protein-protein interactions per pathway versus aggregated retroelement enrichment metrics (NPII_AGG_). Each point corresponds to a pathway.

**FIGURE 4:**
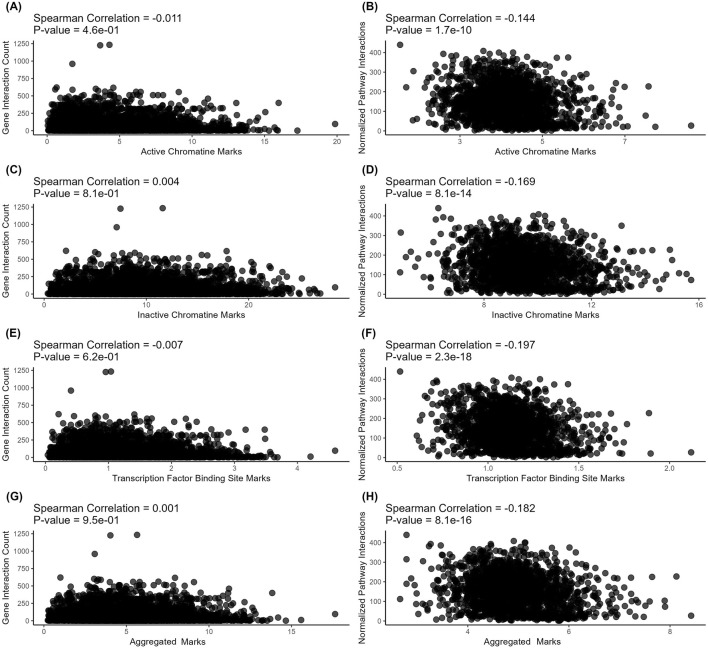
Correlation analysis of gene-level (left) and pathway-level (right) connectivity metrics deduced for *metabolite*-*protein-protein* interactions (all interactions) with the respective *regulatory* evolution rates measured for the active chromatin, inactive chromatin, and TFBS marks. **(A)** Number of all (direct and indirect, with proteins and metabolites) interactions versus active chromatin marks-based retroelement enrichment metrics (NGRE_ac_). Each point corresponds to a gene. **(B)** Averaged number of all (direct and indirect, with proteins and metabolites) interactions per pathway versus active chromatin marks-based retroelement enrichment metrics (NPII_ac_). Each point corresponds to a pathway. **(C)** Number of all (direct and indirect, with proteins and metabolites) interactions versus heterochromatin marks-based retroelement enrichment metrics NGRE_hc_. Each point corresponds to a gene. **(D)** Averaged number of all (direct and indirect, with proteins and metabolites) interactions per pathway versus heterochromatin marks-based retroelement enrichment metrics (NPII_hc_). Each point corresponds to a pathway. **(E)** Number of all (direct and indirect, with proteins and metabolites) interactions versus TFBS-based retroelement enrichment metrics (NGRE_TFBS_). Each point corresponds to a gene. **(F)** Averaged number of all (direct and indirect, with proteins and metabolites) interactions per pathway versus TFBS-based retroelement enrichment metrics (NPII_TFBS_). Each point corresponds to a pathway. **(G)** Number of all (direct and indirect, with proteins and metabolites) interactions versus aggregated retroelement enrichment metrics NGRE_AGG_. Each point corresponds to a gene. **(H)** Averaged number of all (direct and indirect, with proteins and metabolites) interactions per pathway versus aggregated retroelement enrichment metrics (NPII_AGG_). Each point corresponds to a pathway.

On levels of individual genes, we found no meaningful correlations for the connectivity with all types of regulatory evolution rate metrics - for both protein-protein interactions (Figures 3A, C, E, G) and metabolite-protein-protein interactions (Figures 4A, C, E, G). For molecular pathways, we observed a weak negative correlation (from −0.144 to −0.2, Figures 3B, D, F, H; Figures 4 B, D, F, H).

**FIGURE 5:**
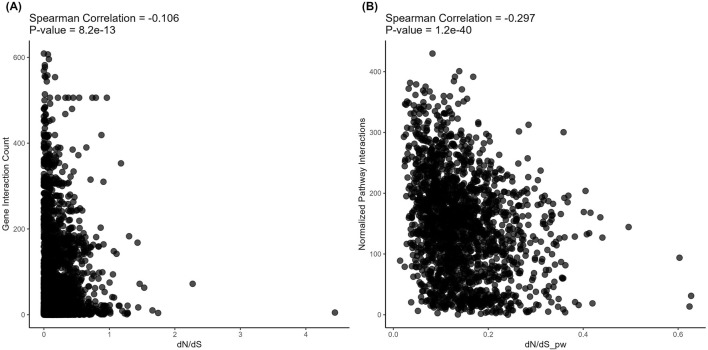
Correlation analysis of gene-level (left) and pathway-level (right) connectivity metrics deduced for *protein-protein* interactions with the respective *structural* evolution rates. **(A)** Number of direct protein-protein interactions versus dN/dS. Each point corresponds to a gene. **(B)** Averaged number of direct protein-protein interactions per pathway versus dN/dS_pw (averaged dN/dS per pathway). Each point corresponds to a pathway.

**FIGURE 6:**
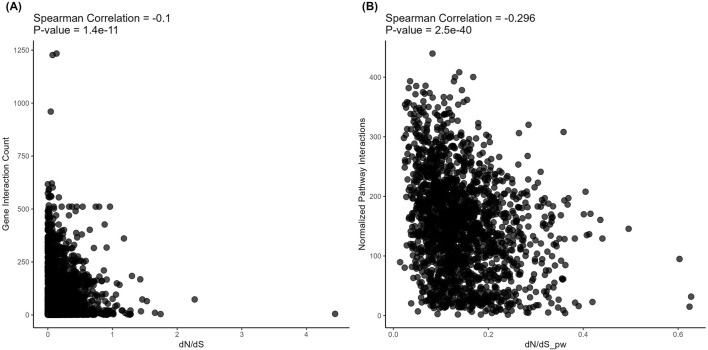
Correlation analysis of gene-level (left) and pathway-level (right) connectivity metrics deduced for *metabolite*-*protein-protein* interactions with the respective *structural* evolution rates. **(A)** Number of all (direct and indirect, with proteins and metabolites) interactions versus dN/dS. Each point corresponds to a gene. **(B)** Averaged number of all (direct and indirect, with proteins and metabolites) interactions per pathway versus dN/dS_pw (averaged dN/dS per pathway). Each point corresponds to a pathway.

However, on the level of *structural* evolution metrics, we detected significant negative correlations with the connectivity for both *protein-protein* (Figure 5) and *metabolite-protein-protein* (Figure 6) interactions. These results are in line with the previously published findings. However, we show here for the first time that in both types of analysis, these correlations were stronger for the pathways than for the individual gene products (Spearman correlation −0.106 vs. −0.297 and −0.1 vs. −0.296, respectively; Figures 5, 6).

**FIGURE 7:**
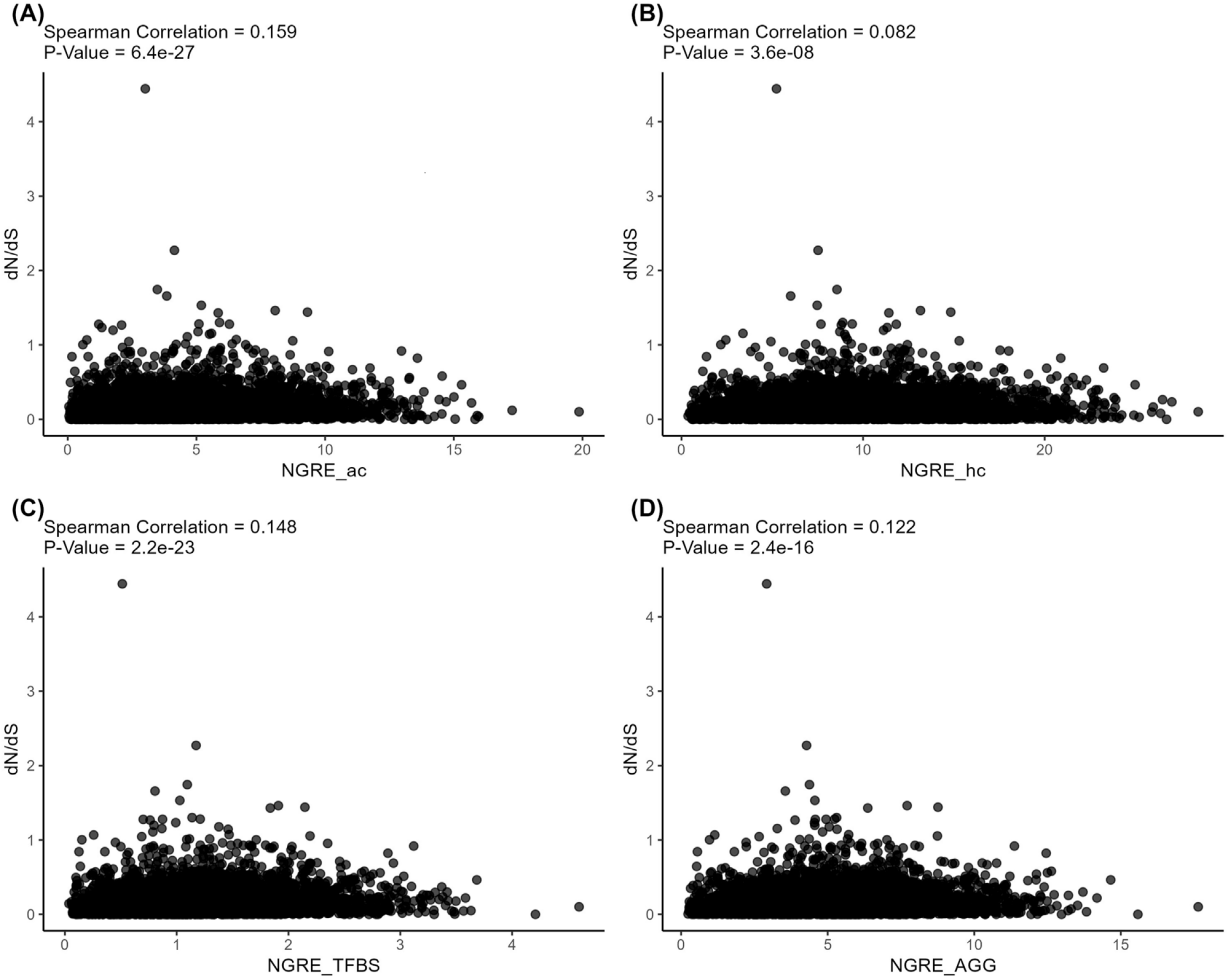
Correlation analysis of *gene-level* structural and regulatory evolution metrics deduced for active chromatin **(A)**, inactive chromatin **(B)**, TFBS **(C)**, and aggregated regulatory evolution metric **(D)**.

**FIGURE 8:**
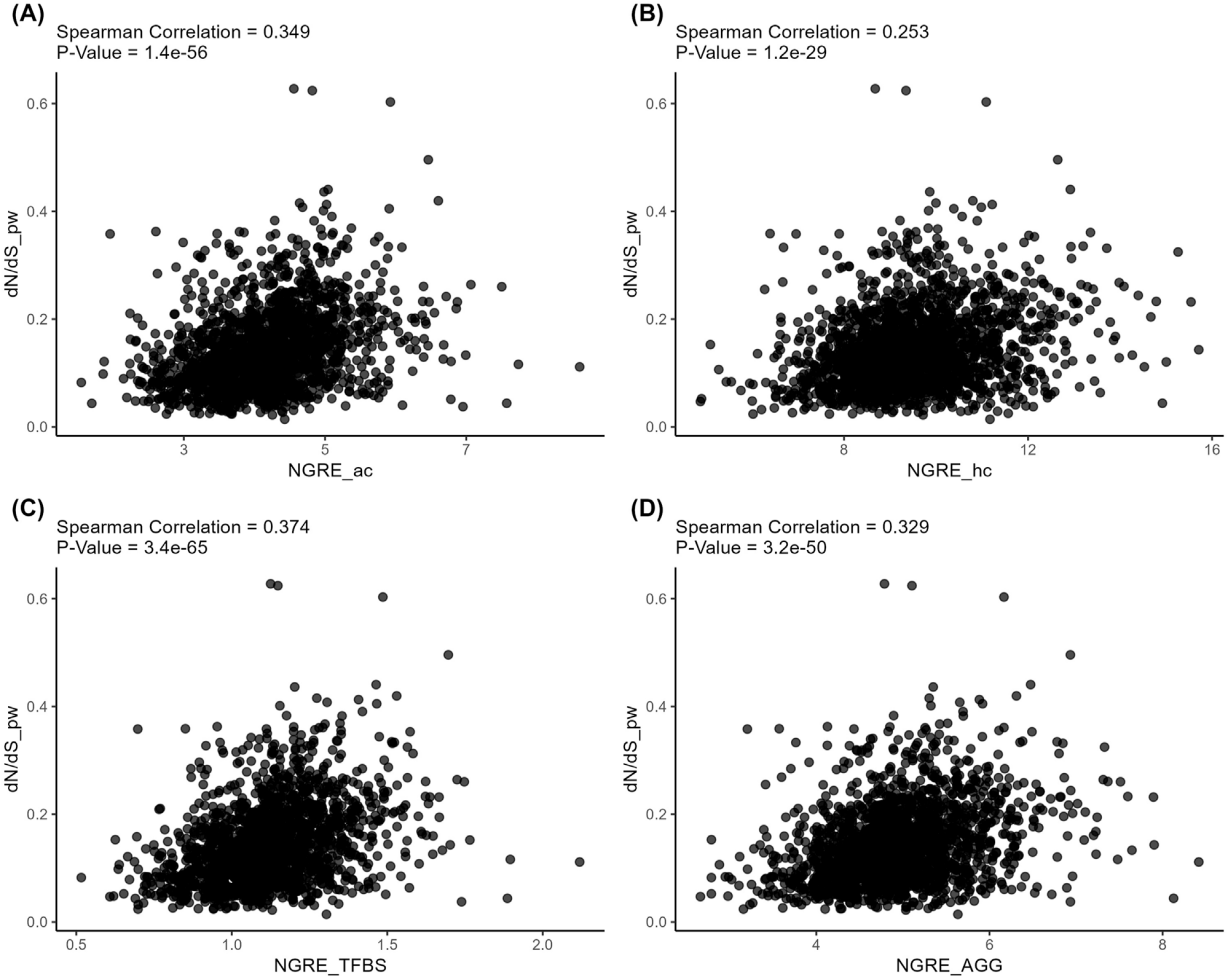
Correlation analysis of *pathway-level* structural and regulatory evolution metrics deduced for active chromatin **(A)**, inactive chromatin **(B)**, TFBS **(C)**, and aggregated regulatory evolution metric **(D)**.

At the same time, the structural and regulatory evolution metrics themselves correlated on both gene (Spearman correlation 0.082–0.159, Figure 7) and pathway (Spearman correlation 0.253–0.374, Figure 8) levels. Again, the pathway level of data analysis resulted in far stronger correlations among the quantitative evolution rate metrics.

## 4 Discussion

Structural and regulatory evolution of protein-coding genes is one of the central areas of evolutionary genomics. The dN/dS ratio, a standard measure of evolutionary pressure, indicates the rate of evolution of protein-coding genes, where values above one indicate positive selection, below one indicates purifying selection, and near one indicates neutral evolution (Kryazhimskiy and Plotkin, 2008). This metric has long been used as a universal barometer of the rate of evolution of genes, highlighting those undergoing rapid evolution or, instead, conservation.

Recently, regulatory changes in gene evolution have attracted increasing attention from the research community. The evolution of gene regulation is often studied through changes in regulatory elements such as transcription factor binding sites (TFBS) and enhancers (Wray et al., 2003). In addition, histone modifications, DNA methylation patterns and transposable elements also play a major role in studying the evolution of gene regulation (Bird, 2007; Feschotte, 2008). The Retrospect method we introduced recently represents a new approach to measure regulatory evolution by quantifying the enrichment of functional motifs in gene promoters, particularly motifs associated with transposable elements. Retroelements (RE), for example, constitute a significant fraction of transcription factor binding sites (TFBS), rearranging the regulatory structure of the human genome. The specific metrics were developed to quantitate the regulatory impact of REs on individual genes with a focus on TFBS and chromatin tags (Nikitin et al., 2019a).

The regulatory context can be extended to the level of molecular pathways and the interactome. Studies integrating evolutionary perspectives have explored the relationship between the evolution of proteins and their properties in interaction networks, such as connectivity (Dosztányi et al., 2006; Mosca et al., 2012), suggesting that there is a complex relationship between a gene’s position in the interactome and its rate of evolution (Hahn and Kern, 2005). It has been repeatedly suggested that proteins with higher connectivity evolve slowly (Brookfield, 2000; Fraser et al., 2002; Fraser et al., 2003; Teixeira et al., 2019). This is probably due to the constraints imposed by their multiple interactions. Lemos et al. (2004) observed a negative correlation between protein-protein interactions and evolutionary diversity in gene expression, implying a possible constraint on the regulatory evolution of genes. This relationship implies that higher levels of protein-protein interactions are associated with reduced variability in gene expression across evolutionary periods, while lower levels of protein interactions may lead to greater variability in gene expression.

In addition, Brown et al. demonstrated that the number of interacting proteins is positively correlated with evolutionary conservation, suggesting that proteins with more interactions are more likely to be conserved (Brown and Jurisica, 2007). In addition, structurally disordered regions of proteins, especially those that play a role in their interaction networks, have been found to be evolutionarily important. As become evident in large-scale analysis of the human, fly, and yeast interactomes, although less conserved, these regions are involved in the evolutionary adaptation of protein networks (Dosztányi et al., 2006; Mosca et al., 2012).

A study of the cancer interactome showed that cancer proteins playing major roles in pathology, evolve more slowly and undergo stronger purifying selection compared to non-cancerous proteins. These proteins show a strong association between their evolutionary age and network connectivity. In these proteins, a significant correlation between nonsynonymous mutation rate and network connectivity was detected, thus highlighting the impact of these mutations on tumor development and progression (Cheng et al., 2014).

We detected a week negative association between the rate of structural evolution and number of protein interactions. The association is strongly statistically significant and may represent a general trend. However, there are remarkable exceptions where the opposite is true, e.g., proteins UBE2U, IL3, and CXCL13 having high dN/dS (>1.3) and at the same time high number of protein-protein interactions (361, 184 and 168, respectively).

On the other hand, regulatory evolution appears to proceed differently than structural evolution. The relationship between the rate of regulatory evolution and position in the interactome is not so clear-cut and may vary depending on specific regulatory elements and mechanisms (He and Zhang, 2006; Jovanovic et al., 2021). So far, different opinions have been expressed as to whether there is a relationship between the rate of protein evolution and the number of protein interactions (Saeed and Deane, 2006). There remains a gap in our understanding of how regulatory evolution is consistent with interactome connectivity.

Here we for the first time investigated relationship between regulatory evolution metrics and number of protein-protein and protein-metabolite interactions. We had no starting hypotheses of whether such an association should exist or not. We observed statistically significant correlation on the level of molecular pathway analysis, but not on the level of individual genes. These results may suggest an overall evolutionary selection trend that largely reshapes the biological processes rather than individual genes.

In our study, we combined quantitative measures of structural and regulatory evolution to analyze the human interactome model built with 7,483 genes. We found a marked correlation between the rates of structural and regulatory evolution of protein-coding genes, evident at both the gene and pathway levels, as assessed by transcription factor binding sites and histone modification mapping data. Our results suggest a common structural/regulatory evolutionary trajectory at the pathway level and weaker but still discernible trends at the gene level.

Weak correlations at the gene level mean that structural and regulatory evolution are relatively dissociated in many individual genes. This may suggest that genes can adopt regulatory flexibility that allows them to change gene expression pattern without altering protein function. Such flexibility may be important for adapting to new environmental or developmental contexts while maintaining the desired protein structure. Other genes may be less conservative structurally but more stable in terms of regulation. Bigger correlations at the pathway level suggest more coordinated evolution because pathways depend on multiple genes working in concert. Significant structural changes in a gene may require regulatory changes to maintain the balance of protein function within a pathway, meaning that coordination between these two forms of evolution becomes more pronounced within integral biological processes.

On the other hand, we detected no significant correlation between the rate of regulatory evolution and human gene connectivity in the interactome model built, suggesting that the dynamics of regulatory mechanisms do not necessarily correspond to the degree of gene connectivity. This indicates that regulatory adaptation may act independently of the frequency of gene interactions.

In this study, we performed the first comparison of structural and regulatory evolution rates with the connectivity, on both gene and pathway levels. A correlation was detected for the structural evolution rates, and the pathway level of data analysis resulted in greater correlations. Thus, as found in several previous studies from different domains (Borisov et al., 2017), the pathway level of data analysis has the advantage of increased data stability.

The correlation between dN/dS and gene connectivity may have applications, for example, for the task of determining the type of inheritance of a gene-related disease. Thus, we verified that both dN/dS and gene connectivity are related to inheritance type. We took genes with known non-conflict inheritance type (543 genes with autosomal dominant inheritance only and 894 genes with autosomal recessive inheritance only accordingly to OMIM) and obtained significant differences in both dN/dS and linkage between genes with different inheritance type (Wilcoxon test p-values of 1.3*10^–38^ and 2.2*10^–13^, respectively, Supplementary Figure S3). Genes with autosomal dominant inheritance were more conservative and had more direct protein-protein interactions than genes with autosomal recessive inheritance. The combination of dN/dS and number of interactions increases the difference (Wilcoxon test p-value 1*10^–40^, Supplementary Figure S3), and can be used as an additional criterion for *in silico* determination of the type of inheritance for gene-related disease. In addition, one of these two parameters can be used when data for another is absent.

We used previously published datasets on human functional gene regulatory markers, which limits the study from capturing novel interactions or evolutionary changes. Nevertheless, we chose data that allowed us to perform a comprehensive assessment of RE-enrichment of histone marks and TFBS. In the future, our analysis can be repeated with greater samples and more diverse types of functional genome marks to further explore the evolutionary trends of human, primate, and non-primate genomes.

This study deepens our understanding of evolutionary interactions at the genetic and pathway levels, offering new perspectives on the adaptive landscape of molecular biology. Our understanding of the co-evolution of structural and regulatory aspects of genes paves the way for further exploration of the complex interdependencies governing gene and pathway evolution, with broad implications for disease research, evolutionary biology, and other fields.

## Data Availability

The original contributions presented in the study are included in the article/Supplementary Material, further inquiries can be directed to the corresponding author.

## Supplementary material

SUPPLEMENTARY TABLE S1 Interactome model, including list of interactions, genes and metabolites.

SUPPLEMENTARY TABLE S2 Lists of genes and molecular pathways analyzed with evolution metrics and number of direct protein-protein and all (protein-protein-metabolite) interactions.

SUPPLEMENTARY FIGURE S1 Selection of the genes and pathways, used in the study.

SUPPLEMENTARY FIGURE S2 Distribution of number of interactions per gene product. **(A)** Number or direct protein-protein interactions. **(B)** Number of all direct and indirect molecular interactions. Red vertical line is threshold for outliers, which were excluded from the study.

SUPPLEMENTARY FIGURE S3 Distribution of number of direct protein-protein interactions and dN/dS for genes with known inheritance type. **(A)** Number of direct protein-protein interactions per gene. **(B)** dN/dS values. **(C)** Combination of dN/dS and number of direct protein-protein interactions (ranked value).
