# Supplementary figures and images for: Number of human protein interactions correlates with structural, but not regulatory conservation of the respective genes
Source: Front Genet. 2024 Oct 29;15:1472638. doi: 10.3389/fgene.2024.1472638 (PMC11554504; doi:10.3389/fgene.2024.1472638)

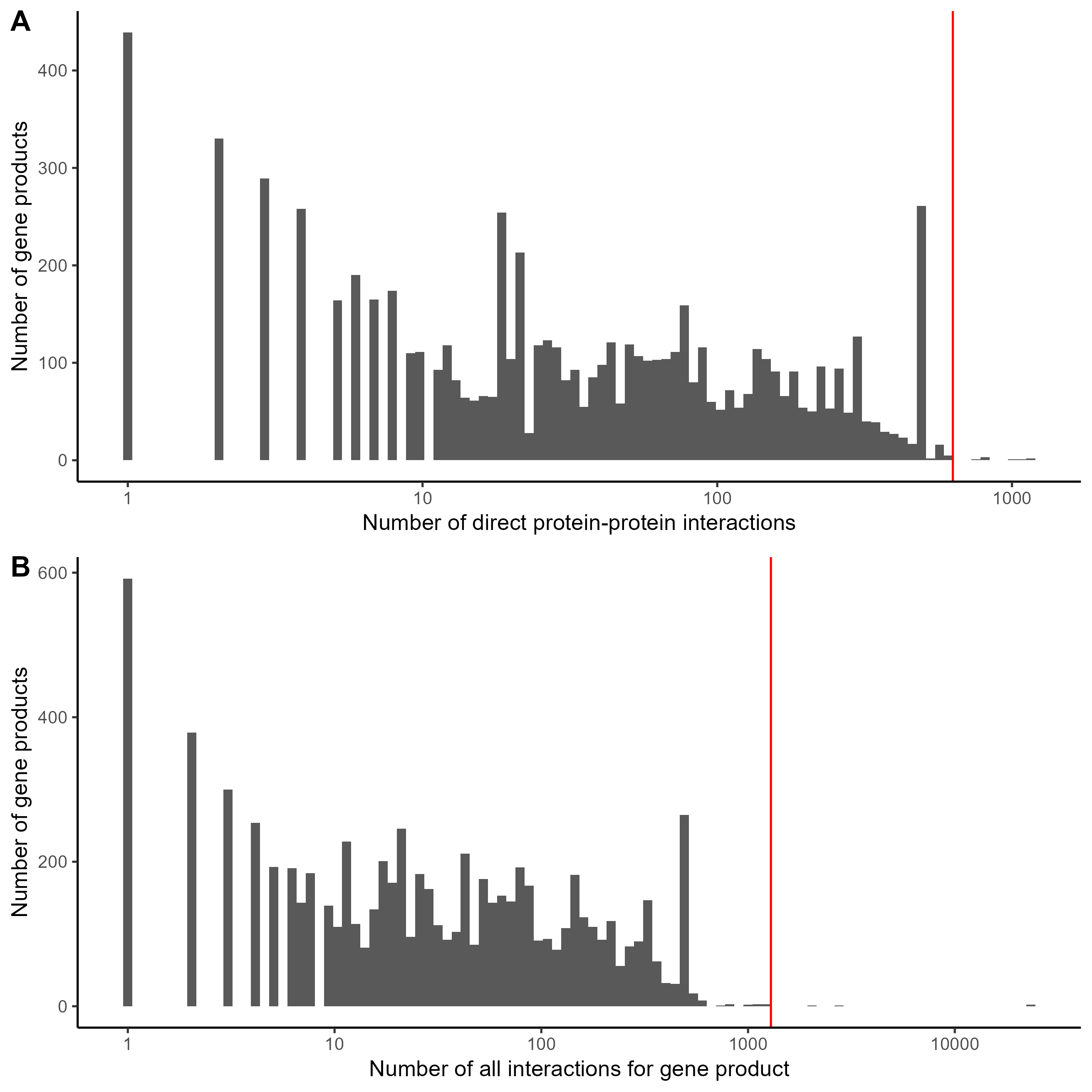

Supplement: Supplementary file 2 [file Image2.PNG]

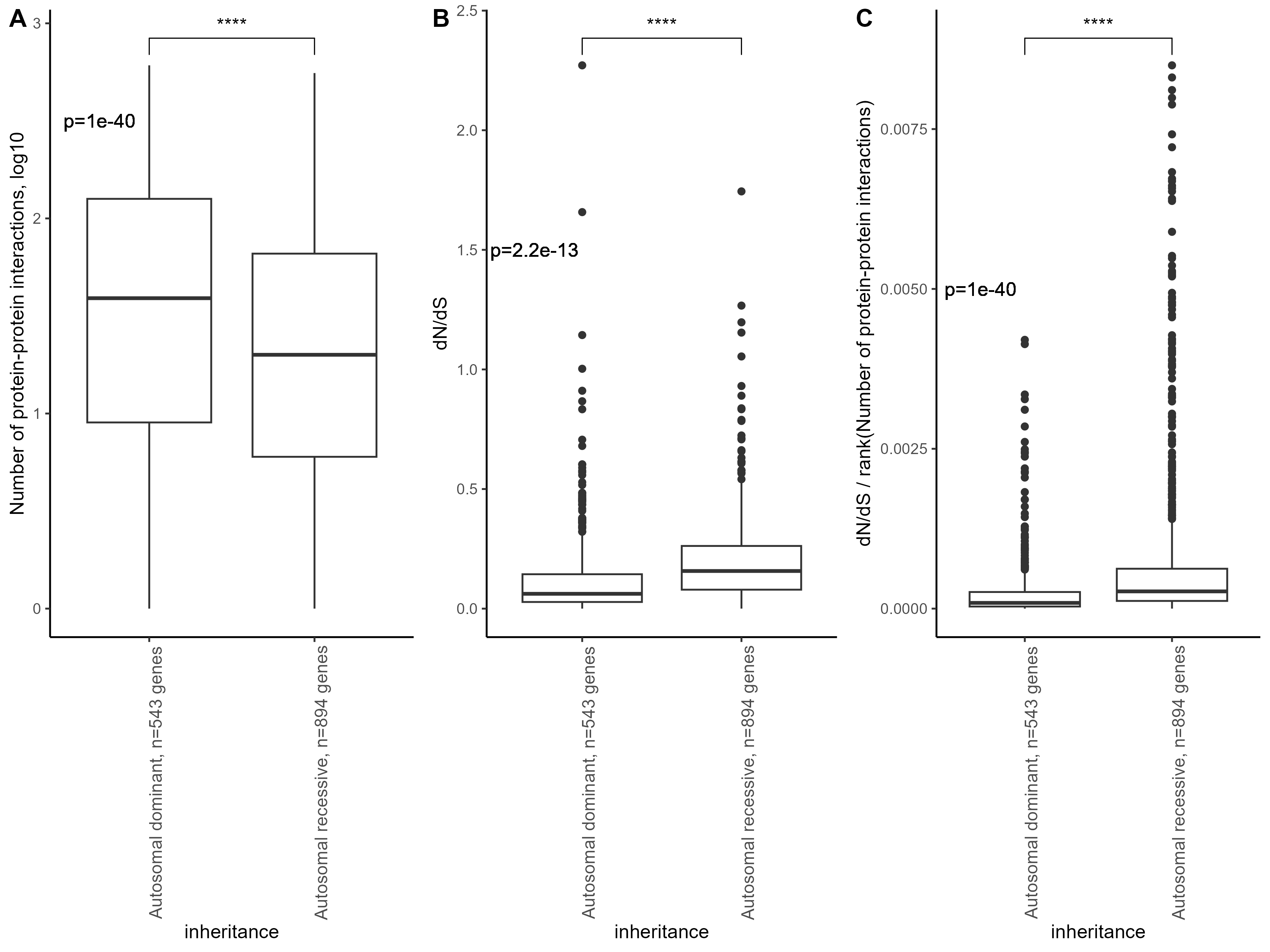

Supplement: Supplementary file 4 [file Image3.PNG]

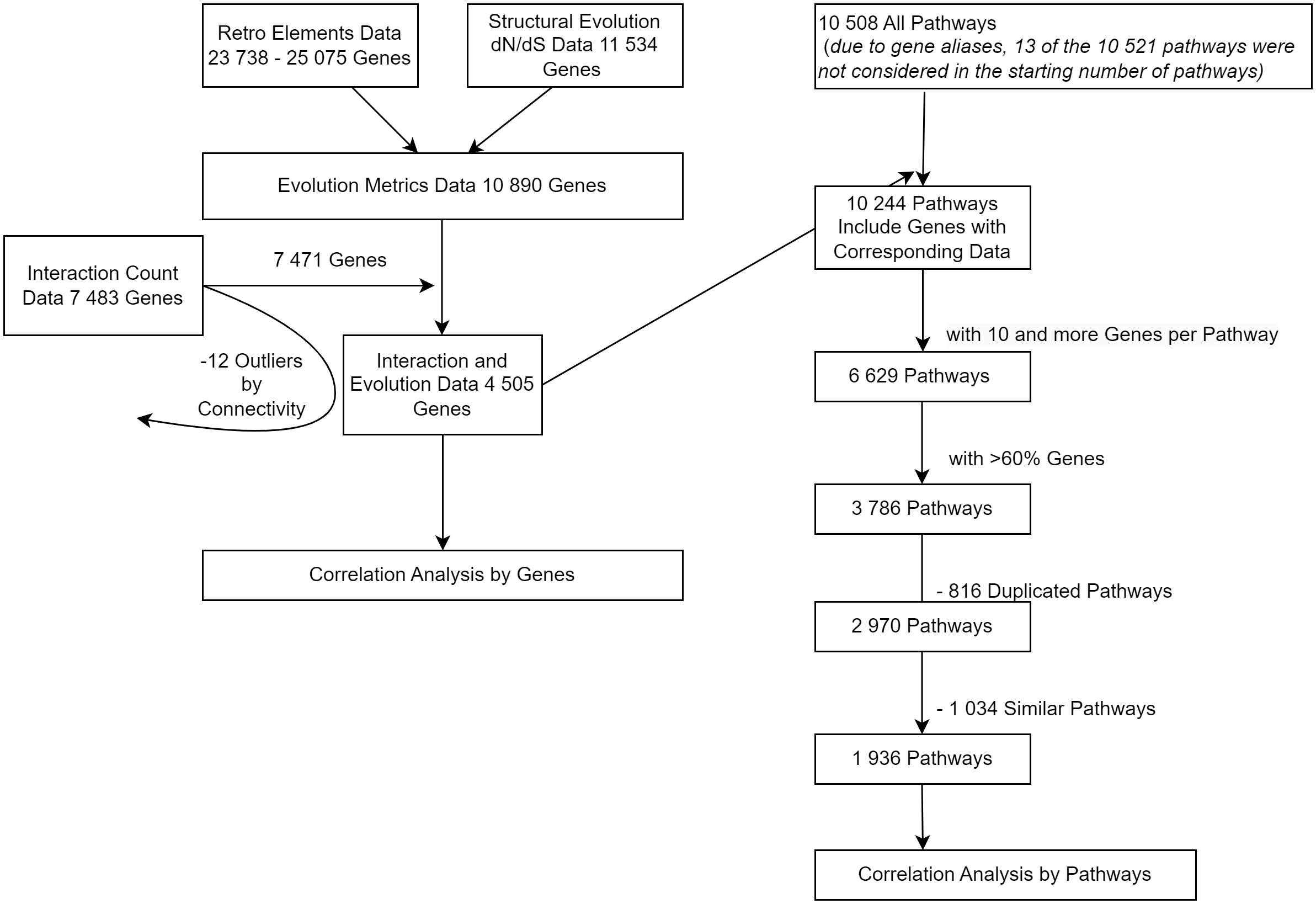

Supplement: Supplementary file 5 [file Image1.jpg]
